# VIOLENCE, IDENTITY, BEHAVIORS, AND ACCESS: MECHANISMS ACCOUNTING FOR CHANGES IN HEALTH OVER TIME OF LGBTQ OLDER ADULTS

**DOI:** 10.1093/geroni/igad104.0706

**Published:** 2023-12-21

**Authors:** Karen Fredriksen-Goldsen, Hailey Jung, Charles Hoy-Ellis, Austin Oswald, Christi Nelson, Hyun-Jun Kim

**Affiliations:** University of Washington, Seattle, Washington, United States; University of Washington, Seattle, Washington, United States; University of Washington, Seattle, Washington, United States; University of Washington, Seattle, Washington, United States; University of Washington, Seattle, Washington, United States; University of Washington, Seattle, Washington, United States

## Abstract

As the population of older adults is growing in complexity and diversity, research has established health disparities by sexual and gender identity among older adults. While most research to date has been cross-sectional, this paper utilizes four-wave longitudinal data from Aging with Pride: National Health, Aging and Sexuality/Gender Study (NHAS) to understand the health and quality of life (QOL) of LGBTQ and sexual and gender diverse older adults over time. Mixed models were run to examine: (1) linear changes over time in health and QOL, controlling for background characteristics, (2) effects of key risk and protective factors on these changes, and (3) gender and age group interactions. Increases in violence, discrimination, identity stigma, adverse health behaviors and experiences (e.g., low physical activity, insufficient nutrition, and suicidal ideation), and decreased access to health and care services were related to poorer health, more cognitive and functional impairment, and lower QOL. Men (vs. women) and the Pride (vs. Silenced and Invisible) Generation were impacted to a greater degree by violence and discrimination, and the impacts of insufficient nutrition and suicidal ideation were also greater in the Pride Generation. Cognitive health of men and gender diverse individuals benefited more strongly from healthcare access. This longitudinal study provides important new findings regarding the mechanisms accounting for the accelerated decline in health and QOL over time in these diverse populations. This information is needed to develop tailored interventions that address these mechanisms as well as are responsive to variations among diverse ages, genders, and sexualities.

